# Rare Case of *Raoultella planticola* Infective Endocarditis after Mitral Valve Replacement Surgery

**DOI:** 10.3390/diseases11040133

**Published:** 2023-09-29

**Authors:** Diana Roxana Opriș, Victor Vacariu, Alexandru Petru Ion, Timea Szigyarto, Emil Marian Arbănași, Eliza Russu, Maria Mihaela Opriș

**Affiliations:** 1 Emergency Institute of Cardiovascular Diseases and Transplantation (IUBCVT), 540136 Targu Mures, Romania; 2 George Emil Palade University of Medicine, Pharmacy, Science and Technology of Targu Mures, 540139 Targu Mures, Romania; 3 Clinic of Vascular Surgery, Mures County Emergency Hospital, 540136 Targu Mures, Romania; emil.arbanasi@umfst.ro (E.M.A.);; 4 Doctoral School of Medicine and Pharmacy, George Emil Palade University of Medicine, Pharmacy, Science and Technology of Targu Mures, 540139 Targu Mures, Romania; 5 Department of Vascular Surgery, George Emil Palade University of Medicine, Pharmacy, Science and Technology of Targu Mures, 540139 Targu Mures, Romania

**Keywords:** infective endocarditis, rare bacteria, *Raoultella planticola*, mitral valve prosthesis

## Abstract

Infective endocarditis remains a condition associated with high morbidity and mortality, regardless of advances in diagnosis and therapeutics. The etiology, microbiology, and epidemiology of infective endocarditis have changed in the last years, with healthcare-associated infective endocarditis being responsible for a myriad of cases. *Raoultella planticola* is rarely the cause of infective endocarditis. We present a 72-year-old Caucasian female with a history of mitral valve replacement for rheumatic valve disease two months before the current presentation, without any immunosuppressive pathologies, diagnosed with *Raoultella planticola* infective endocarditis. Long-drawn antibiotic treatment led to a full recovery with no evidence of recurrence or relapse. This report highlights the importance of a multimodal approach for the diagnosis of bacterial etiology, the importance of selection and duration of an appropriate antibiotic regimen, and the presence of a rare opportunistic bacteria that has proven pathogenicity in a wide range of organ systems, usually in patients with several risk factors.

## 1. Case Presentation

**Figure 1:**
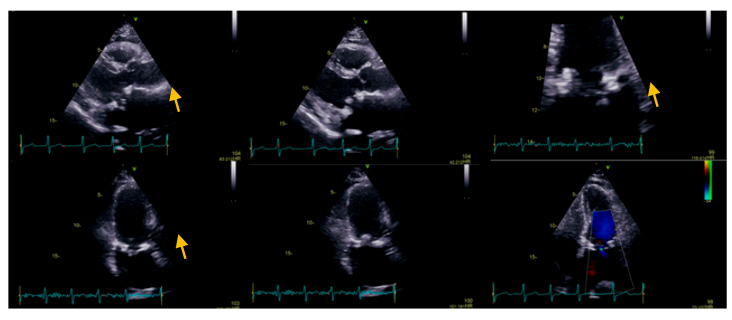
Transthoracic echocardiography: parasternal long axis view, the zoomed image on mitral valve (apical four-chamber view), and the apical two-chambers view, the yellow arrow showing a mobile, isoechoic, homogeneous filiform mass of 10 mm, attached to the mitral prosthetic valve, with minor intra-prosthetic regurgitation.

We present the case of a 72-year-old Caucasian female who presented for dyspnea and fatigue at moderate intensity activity, a two-week history of intermittent fever (which decreased after administration of antipyretic), perspiration, loss of appetite, and weight loss (4 kg in the last two months). Her medical history includes mitral valve replacement for rheumatic valve disease, with a biological prosthesis (Labcorp No 29) two months before the current presentation. On admission, the patient was afebrile, with a heart rate of 110 b.p.m., blood pressure of 110/80 mmHg, and oxygen saturation of 98% on room air. The physical examination revealed no skin pallor or rashes, post-sternotomy scar, no adenopathy, the chest was clear to auscultation bilaterally, normal jugular venous pressure, tachycardic heart rate with normal heart sounds, no murmurs, normal pulses, no peripheral edema, well-perfused peripheries, and unremarkable findings regarding other systems. At the presentation, the electrocardiogram showed atrial tachycardia with variable conduction (2:1, 3:2), 110 b.p.m., normal QRS axis, without any conduction disturbances (PR 160 ms, QRS 80 ms), and unremarkable repolarization features. On the transthoracic echocardiography, we noticed a dilated left atrium (108 mL, 56 mL/m^2^), a normal left ventricle with normal systolic function with a 3D derived left ventricle EF of 50%, and global longitudinal strain −20.1%. Regarding right chambers, we observed a normal right atrium, normal size of the right ventricle, with slightly reduced longitudinal function, tricuspid annular plane systolic excursion (TAPSE) of 15 mm, respectively, and Tissue Doppler imaging (TDI)-derived tricuspid lateral annular systolic velocity (S’) of 9 cm/s (common post-cardiac surgery), despite a fractional area change (FAC) of 38% and 3D-derived right ventricle EF of 46%. As regards the valvular apparatus, we noticed a mobile, isoechoic, homogeneous filiform mass of 10 mm attached to the mitral prosthetic valve, with minor intra-prosthetic regurgitation, minor aortic regurgitation, and mild tricuspid regurgitation, without pulmonary hypertension (Figure 1).

**Figure 2:**
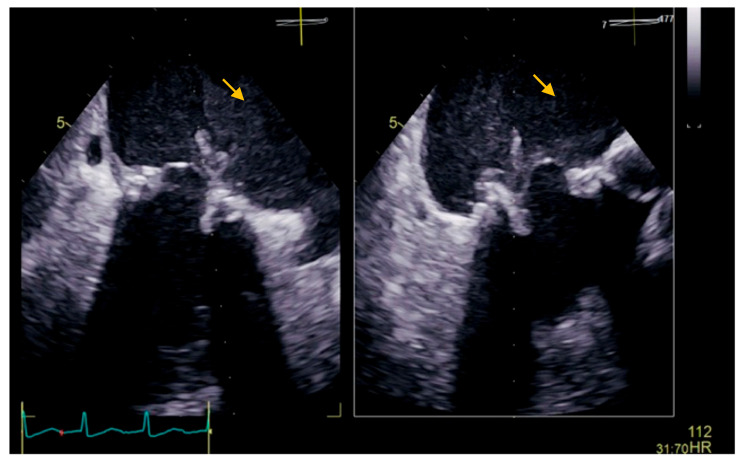
Transesophageal echocardiography showing a biological prosthesis in the mitral position, with two mobile, isoechoic, homogeneous filiform masses of 10 mm attached to the posterior portion of the prosthesis on the atrial surface (yellow arrow).

The transesophageal echocardiography showed a biological prosthesis in the mitral position, with a mean diastolic trans-prosthetic gradient of 7 mmHg, with two mobile, isoechoic, homogeneous filiform masses of 10 mm, attached to the posterior portion of the prosthesis, on the atrial surface, causing a minor intra-prosthetic regurgitation, with no thrombi in the left atrium auricula (Figure 2).

Laboratory findings reflected a nonspecific acute inflammatory response, manifested as an increased erythrocyte sedimentation rate (ESR 60 mm/h), elevated C-reactive protein (CRP 1.35 mg/dL), elevated fibrinogen (514 mg/dL), normal white blood cell count and platelet count and normochromic normocytic anemia (Hb 9.5 g/dL) with normal ferritin. Normal renal (glomerular filtration rate-GFR 80 mL/min/1.73 m^2)^ and hepatic function (Table 1).

**Figure 3:**
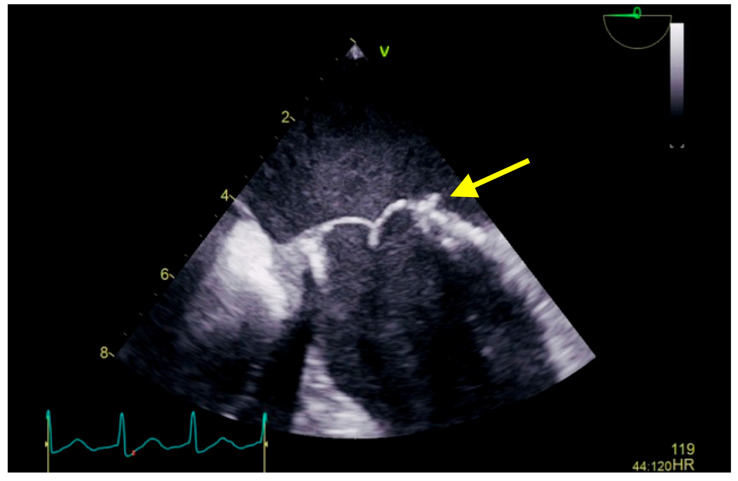
Transesophageal echocardiography showing a biological prosthesis in the mitral position, yellow arrow showing a reduced dimensions of the vegetation compared to the previous examination. A 6 months follow-up revealed stationary aspect.

Raising the suspicion of endocarditis of the prosthetic valve (considering patient symptoms: two-week history of intermittent fever, perspiration, loss of appetite, and weight loss; the mass attached to the mitral prosthetic valve; and laboratory findings), we collected three sets of blood culture, at three different venipuncture sites, that isolated *Raoultella planticola*, sensitive to *ampicillin*, *cefuroxime*, and *gentamicin*. Bacterial isolates were inoculated into the appropriate VITEK 2 Compact identification; afterward, data obtained were analyzed using VITEK 2 software version 9 in accordance with the manufacturer’s instructions. With definite infective endocarditis based on modified Duke’s Criteria, according to the antibiogram, in consultation with an infectious disease specialist, we initiated antibiotic treatment with intravenous *cefuroxime* 1.5 three times a day for the following 25 days. The patient evolution was favorable; without febrile episodes, laboratory results showed a decline in inflammatory parameters. Before discharge, the echocardiographic evaluation (transthoracic as well as repeated transesophageal echocardiography) revealed a reduction in the size of vegetation compared to the previous examination (Figure 3), and the repeated blood cultures were negative. At discharge, she continued the antibiotic treatment with *cefuroxime* per os two times a day for the following 6 weeks. After 6 months, follow-up showed no evidence of recurrence or relapse; clinical, laboratory (negative inflammatory parameters and negative blood culture), and imagistic (transthoracic and transesophageal) assessments were performed.

## 2. Discussion

Infective Endocarditis represents a condition that involves multiple systems and results from infection of the endocardial surface of the heart [1]. Prosthetic valve endocarditis is a type of infected endocarditis represented by microbial infection of the endovascular part of a prosthetic valve or on the reconstructed native valve of the heart as well as an indwelling cardiac device [1]. Even nowadays, the diagnosis of infective endocarditis remains challenging, given the diverse nature and evolving epidemiological characteristics of infective endocarditis. Modified Duke criteria (published in 2000, first proposed as Duke criteria by Durack and associates in 1994) is currently the most specific and sensitive method for diagnosis [2]. It provides a more standardized approach for diagnosis considering microbiological analysis, aspects of multimodality imaging, and clinical features. Applying these criteria, the probability of infective endocarditis can be expressed as *definite*, *possible,* and *rejected*. For *definite* infective endocarditis, two main criteria, one major criterion and three minor criteria, or five minor criteria must be met. The diagnosis of infective endocarditis is labeled as *possible* when there is fulfilled one major criterion and one minor criterion or three minor criteria. Respectively, the diagnosis of infective endocarditis may be *rejected* when there is a firm alternate diagnosis; the resolution of symptoms suggests infective endocarditis with antibiotic therapy for equal or less than four days; there is no pathologic evidence of infective endocarditis at surgery or autopsy, or in the case of the criteria as stated in the case of a possible diagnosis of IE not being met [3]. In consideration of the miscellaneous clinical presentation of infective endocarditis, the modified Duke criteria should be applied after judicious clinical judgment. 

Even though numerous improvements regarding diagnostic and therapeutic strategies have been carried out, infective endocarditis remains a disease associated with high mortality and morbidity [4,5,6,7,8]. Several causes for the persistently poor prognosis have been revealed in the literature [4] and include an increased proportion of patients with prosthetic valves or device-related infection, an aging population with more severe disease, and increasing nosocomial cases in recent decades have been described [4,9]. Infective endocarditis is a relatively common infection that occurs in immunocompromised patients, especially in those with a cardiovascular substrate, and is usually caused by the Staphylococcus, Enterococcal, and Streptococcus species. The availability of more precise identification techniques has substantially extended the spectrum of bacteria incriminated in the etiologic diagnosis of endocarditis. *Raoultella planticola* is rarely the cause of infective endocarditis.

To provide an optimal antimicrobial therapy, it is essential not only to diagnose infective endocarditis but also to obtain an etiologic diagnosis, to establish which antibiotherapy should be used based on blood culture and drug susceptibility tests, and to determine bactericidal regimen of proven efficacy [2]. For proper therapeutic management of this complex disease, a team approach (cardiologist, specialist trained in infectious disease, and cardiothoracic surgeon) is recommended. Antibiotics are the mainstay of medical therapy, although, in some cases of complicated infective endocarditis, a surgical approach is needed.

*Raoultella planticola* is broadly considered a harmless bacteria, gram-negative, aerobic, non-capsulated, non-motile rods, frequently isolated from natural environments with high prevalence in soil, water, herbs, and fresh vegetables. First described by Freney et al. in 1984 in a patient with sepsis, known as a member of the *Klebsiella* genus, reclassified in 2001 taking into account 16SrRNA and rpoB gene sequencing [10]. *Raoultella planticola* is capable of producing histamine by the decarboxylation of histidine, a process induced by enzymes produced by the aforementioned microorganism, which can be found in the fish’s cutis and intestines and can lead to symptoms of scromboid poisoning after ingesting large amounts of poorly prepared seafood [11]. *Raoultella planticola* is an opportunistic microorganism that can develop different infections of the biliary tract, respiratory system, urinary tract, and even bacteremia in immunocompromised patients. Several pathologies such as dialysis, diabetes melitus, malignancy, immunocompromised states, and certain medical therapies led to an increased risk of infection. Several studies found that 9–18% of the population is colonized with the aforesaid bacteria, mainly in the digestive tract or the upper respiratory tract, but they can also survive in hospital environments [12]. There are many resemblances due to the close phylogenetic relationship between *Raoultella planticola* and *Klebsiella* spp. [13], and it is supposed that both of these species behave alike within their host, react similarly to antibiotherapy, and have the same capacity to develop drug resistance [12,14,15,16].

The epidemiology of *Raoultella planticola* infections is unclear since the availability of surveillance studies is scarce in the literature due to difficult differentiation from *Klebsiella* species; consequently, the incidence could be underestimated. Over the last few years, the diagnostic rate has been refined, which led to an increased number of case reports; hence, *Raoultella planticola* should be considered as precedently underdiagnosed instead of rare or emerging microorganisms [13]. The clinical presentation of *Raoultella planticola* infective endocarditis is similar to the presentation of infective endocarditis secondary to other bacteria. The patient discussed above presented in our clinic for two months postoperative follow-up and complained of various symptoms such as dyspnea and fatigue at moderate intensity activity, intermittent fever in the last month, perspiration, inappetence, and weight loss of 4 kg, symptomatology installed after mitral valve replacement. Echocardiographic examination revealed two mobile, isoechoic, homogeneous filiform masses of 10 mm attached to the posterior portion of the prosthesis on the atrial surface, raising the suspicion of infective endocarditis on the prosthetic valve. We collected three sets of blood cultures at three different venipuncture sites, which identified the incriminate pathogen as *Raoultella planticola*. The development of more precise microbial identification and characterization tools used for the differentiation of *Klebsiella* spp. and *Raoultella* spp. was an important step toward a prompt and accurate diagnosis. Susceptibility tests allowed an appropriate therapy to be implemented, thereby improving prognosis. Generally, *R*. *planticola* is sensitive to a wide range of antibiotics, though, akin to *Klebsiella* spp., *R*. *planticola* can acquire plasmid-borne antibiotic-resistance genes, causing severe and even fatal infections. Definitive diagnostic of infective endocarditis was based on the modified Duke’s Criteria—*one major* criterion (echocardiography evidence of vegetation on mitral valve biological prosthesis) and *three minor* criteria (fever, mitral valve replacement as a predisposing heart condition and positive, repeated blood culture for *Raoultella planticola*).

Optimal management of infective endocarditis is based on an adapted and prolonged antibiotic regimen combined with surgical excision in selected patients. Although the frequency with which surgery management of this disease increased in the last decades (approximately one-half of patients with infective endocarditis) with a concomitant decrease in early mortality, the type and the timing of surgery are still debated and may vary among different centers [2]. The current practice guidelines recommend that surgery should be considered as a therapy for complicated infective endocarditis such as *severe congestive heart failure* secondary to acute left-sided valvular regurgitation; *uncontrolled infection*, particularly in case of persistent fever and positive blood culture for more than 7–10 days despite optimal antibiotic management frequently associated with several anatomic findings, such as abscess formation, constitution of fistulas or false aneurysms, or increasing vegetation size; respectively in case of characteristics suggestive of a high risk of *embolic events* [2,3]. Surgery is recommended if a large, mobile vegetation (more than 10 mm) is present, especially after an embolic event occurred despite optimal antibiotic treatment. Furthermore, an indication for an early surgery approach represents the presence of heart failure, severe valvular malfunction, or abscess added to a large vegetation (more than 10 mm) [2,3]. However, regarding our case, none of these specific indications were present, the favorable course being obtained after an appropriate antibiotic regimen.

Multiple studies reported that *Raoultella planticola* is susceptible to antibiotics frequently used for infections caused by *Enterobacteriaceae*, although it has been reported their resistance to *penicillins* and *ampicilins* [13] Antibiotherapy should be established on blood culture and drug susceptibility tests. Uncommonly, in our case, the antibiogram revealed sensitivity to *ampicillin*, *cefuroxime*, and *gentamicin*. Considering antibiogram results, patient age, and published materials, we decided to initiate intravenous monotherapy with *cefuroxime* for the following 25 days and continued afterward with *cefuroxime* per os (two times per day) for the following 6 weeks, with a favorable outcome.

An important aspect of almost all patients reported to be infected with *Raoultella planticola* is the immunocompromised status. The main risk factors described are premature, prolonged hospitalization in ICU, long-term antibiotic therapy, enteral feeding tubes, diabetes mellitus, chronic renal dysfunction, cancer and chemotherapy, steroid use, and catheters [10,17].

We have presented a rare case of *Raoultella planticola* infective endocarditis in a patient without any immunosuppressive pathologies who underwent mitral valve replacement two months before the current presentation. It is universally accepted that cardiac surgery with cardiopulmonary by-pass promotes not only systemic pro-inflammatory status as a consequence of the aggregation of different factors such as surgical stress, ischemia-reperfusion injury, activation of blood components in the extracorporeal circuit, endotoxin release and activation of immune cells but also generates a mild immunosuppression state [18,19,20,21]. However, we presume that the infection was nosocomial in a patient who underwent cardiac surgery, the only factor that could weigh in on the immunosuppressive status, although the source of the pathogen is unclear. The presence of a foreign body (mitral valve prosthesis), ICU stay, and the presence of indwelling catheters were additional risk factors. To our knowledge, there are only two reported cases of *Raoultella planticola* causing IE, one of them in a pediatric patient [22] and the second one in a patient undergoing hemodialysis [23].

## 3. Conclusions

The etiology, microbiology, and epidemiology of infective endocarditis are undergoing a period of change, with the development of several new risk features, respectively, with an increasing incidence of nosocomial endocarditis and cardiovascular implantable electronic device infections (CIEDs).

The presented case is, to our knowledge, the third case of *Raoultella planticola* infective endocarditis, an opportunistic bacterium, usually misdiagnosed as *Klebsiella* spp., which has proven pathogenicity in a wide range of organ systems, usually in patients with several risk factors. The peculiarity of the presented case is an early postoperative infection caused by *Raoultella planticola* in a patient without other immunosuppressive conditions, which led to bacteremia and infective endocarditis. *Raoultella planticola* should be considered as a potential cause of surgical site infection in postoperative status, even in patients without additional reasons for immunosuppressive status, patients who are more prone to develop infections with low virulent organisms. Although *Raoultella planticolla* is susceptible to various antibiotics frequently used, a diversity of acquired genes leading to resistant strains has been reported in the last years. Therefore, antibiotic management should be carried out according to drug susceptibility tests after proper identification of incriminating microorganisms. An aggressive therapeutic strategy in high-risk patients is necessary to reduce the morbidity and mortality associated with this disease.

## Figures and Tables

**Table 1:** Laboratory tests and results.

Laboratory Test	Results			Reference Range
	Admission	Before Discharge	6 Months Follow-Up	
White blood cell count	7060	6800	7000	3600–10,000/uL
Hemoglobin	9.5	10.5	12.6	11–15 g/dL
Platelet count	246,000	317,000	300,000	150,000–450,000/uL
C-reactive protein	1.35	0.47	0.27	0–0.5 mg/dL
Fibrinogen	514	380	350	30–400 mg/dL
Erythrocyte sedimentation rate	60	30	15	5–17 mm/h

## Data Availability

The data that support the findings of this study are available from the corresponding author upon reasonable request.
